# Actions and the Self: I Give, Therefore I am?

**DOI:** 10.3389/fpsyg.2021.684078

**Published:** 2021-08-10

**Authors:** Tobias Regner, Astrid Matthey

**Affiliations:** ^1^Department of Economics, University of Jena, Jena, Germany; ^2^Economic and Social Environmental Issues, Max Planck Institute of Economics, Jena, Germany; ^3^Umweltbundesamt/German Environment Agency, Dessau-Roßlau, Germany

**Keywords:** social preferences, pro-social behavior, moral wiggle room, self-image concerns, self-signaling, reciprocity, experiments

## Abstract

Self-signaling models predict less selfish behavior in a probabilistic giving setting as individuals are expected to invest in a pro-social identity. However, there is also substantial evidence that people tend to exploit situational excuses for selfish choices (for instance, uncertainty) and behave more selfishly. We contrast these two motivations (identity management and self-deception) experimentally in order to test which one is more prevalent in a reciprocal giving setting. Trustees' back transfer choices are elicited for five different transfer levels of the trustor. Moreover, we ask trustees to provide their back transfer schedule for different scenarios that vary the implementation probability of the back transfer. This design allows us to identify subjects who reciprocate and analyze how these reciprocators respond when self-image relevant factors are varied. Our results indicate that self-deception is prevalent when subjects make the back transfer choice. Twice as many subjects seem to exploit situational excuses than subjects who appear to invest in a pro-social identity.

**JEL classifications**: C72, C91, D80, D91

## 1. Introduction

Many people behave pro-socially—if the only other choice is selfish behavior. But what if the situation is less transparent? What if circumstances exist that allow a selfish choice while simultaneously a pro-social image in front of one self can be kept? Dana et al. (2007) find that giving rates are significantly reduced when moral excuses for selfish behavior are available. People seem to make use of ‘moral wiggle room', a term coined by them, and evidence from a series of studies (e.g., Larson and Capra, 2009; Haisley and Weber, 2010; Hamman et al., 2010; Matthey and Regner, 2011; Feiler, 2014; Grossman, 2014; van der Weele, 2014; Exley, 2015) confirms such a self-serving bias in dictator game giving.

Bayesian self-signaling models propose a different type of behavior in an allocation decision. Individuals derive utility the more they believe they are a pro-social type. However, they are inherently unsure whether they actually are a pro-social type (or instead pro-self). Thus, they may give in order to send a positive signal to their self. Grossman (2015) develops such a model and tests it experimentally in a binary probabilistic dictator game. The model predicts more giving in the low probability treatment as the expected cost of sending a pro-social signal is cheapened. However, results do not lend supporting evidence.^1^

Bénabou and Tirole (2011) consider further channels how actions and the self relate to each other. Besides identity management (beliefs about one's type are malleable through actions) they also allow, for instance, for the possibility that an action may be regarded as uninformative about the self. Hence, individuals may attribute their selfish action to the context, instead of having to connect selfish behavior to their self-image. The salience of the context—to what extent one's action has an effect on the outcome—systematically varies the informativeness of an action. As a consequence, individuals would have a higher tendency to invest in their identity, when informativeness is high. Likewise, they would tend to succumb to the temptation of situational excuses, when informativeness is low. Naturally, it is in the self's ‘eye of the beholder', whether an action is perceived to convey information about one's underlying character (potentially leading to an identity investment) or whether situational excuses are invoked and used to bias the signal to the self (making the identity damage of a selfish choice acceptably small). Thus, it is an empirical question which motivation is more dominant. Our study's goal is to consider both self-signaling channels (identity management and self-deception), test them experimentally, and thus shed more light on their relative prevalence.

We set up a probabilistic giving environment in which the predictions of the two approaches contrast each other. Identity management predicts higher transfers with increased uncertainty about the implementation of the transfer due to the pro-social signal becoming cheaper. Instead, self-deception predicts lower transfers, because uncertainty about the actual implementation could serve as a situational excuse.

Since the effect of moral wiggle room on giving in dictator games is well established, we decided to move our test bed to a less explored domain, namely, when also reciprocal concerns may motivate individuals^2^. For this purpose we conduct a modified trust game. Trustees' back transfer choices are elicited for five different transfer levels of the trustor. Moreover, we ask trustees to provide their back transfer schedule for different scenarios. While in scenario 1 the back transfer will be implemented for sure, in scenarios 2–4 there is a positive probability that the back transfer fails. In such a case the trustee gets to keep the available amount. After trustees have chosen their back transfer schedules for all scenarios, they are informed that they can select the scenario they would like to get implemented.

This design allows us to identify subjects who reciprocate (based on the back transfer schedule in scenario 1) and analyze how these reciprocators behave. Two situational excuses for selfish behavior are present in our design. First, the fact that in scenarios 2–4 the transfer could fail may serve as an excuse to return less in these scenarios (alternatively, the decreased implementation chance of the transfer could induce subjects to return more via identity management). Second, having to choose a scenario can imply the temptation of picking a favorable scenario—one that results in a monetary gain (in expectations)—while the trustor might not receive anything.

Our within-subjects design allows us to analyze back transfer choices at the individual level. Thus, we can distinguish between trustees motivated by self-deception and identity management. While our results show that behavior consistent with self-deception is more common when subjects make the back transfer choice (twice as many subjects decrease than increase their transfers under uncertainty), they also indicate that both motivational processes appear relevant for human decision making. Furthermore, as a substantial fraction of subjects makes a self-serving scenario choice, our results indicate that reciprocators make use of moral wiggle room if situational excuses exist.

The paper is organized as follows. In section 2 we describe the experiment and present behavioral predictions. Results are reported and discussed in section 3. We conclude in section 4.

## 2. Experiment

### 2.1. Design

The experiment consisted of a variant of the trust game (Berg et al., 1995). Both trustor and trustee received an endowment of 10 Euro. As the first step, the trustor could send either 0, 2.50, 5, 7.50, or 10 Euro to the trustee. This transfer was tripled and added to the trustee's account, who could then return any amount available on the account to the trustor. That is, depending on the trustor's transfer trustees could return up to 10, 17.50, 25, 32.50, or 40 Euro. All subjects played in both roles. They knew that it was determined randomly at the end of the experiment whether a subject acted as trustor or trustee. Trustees' decisions were elicited using the strategy method, that is, a trustee decided how much to send back to the trustor for all possible transfers. Hence, all trustees made five back transfer decisions, one of which was to become relevant according to the trustor's actual transfer. When entering their back transfer choices, trustees were informed about the respective amount they would receive at each transfer level. Trustors only learned the outcome, not the choice of the trustee.

Trustees knew that they make the back transfer choices for different scenarios. In scenario 1, the trustee's transfer was carried out with certainty, that is, it reached the trustor for sure and was subtracted from the trustee's account. In scenario 2, the transfer was carried out with 90% probability. With the remaining 10% probability, the trustee would keep the available amount. In this case, the trustor would be left with her endowment minus the amount she sent to the trustee, independently of the size of the trustee's back transfer. In scenario 3, the trustee's transfer was carried out with 80% probability, with 20% the trustee kept the entire amount. In half of our sessions we added a fourth scenario in which the trustee's transfer was or was not carried out with equal probability. Scenario 4 was employed to test whether the availability of an option with a much smaller transfer probability would serve as an excuse to choose a scenario with a transfer probability below 1 (but above 50%) rather than the certain transfer.

Overall, subjects therefore made five back transfer decisions (for each possible amount sent by the trustor) per scenario. After trustees completed all choices for one scenario, they were asked for their back transfers in the next scenario. Choices from previous scenarios were still visible. Supplementary File 1 shows a screenshot of the decision interface for the scenario 1 choice and (Supplementary File 1) one for the scenario 3 choice when a subject has already entered back transfers for scenarios 1 and 2. It illustrates the sequential nature of entering the back transfer schedules for the scenarios and the fact that subjects were reminded of their choices in previous scenarios. We chose to provide choices in all previous scenarios in case a subject would like to take the same decision across scenarios. Since not just one decision but an entire back transfer schedule consisting of five choices would have to be remembered, we decided the interface should provide a reminder.

Subjects were instructed that the scenario to be implemented “would be decided” after they made all choices. No specific decision mechanism was mentioned. After subjects had made all decisions, they were shown an overview screen with their transfers for all scenarios. They were informed that they could choose themselves which scenario they wanted to apply. At this point, the uncertainty about the back transfer implementations had not yet been resolved, thus, subjects still did not know whether their chosen back transfer would occur or not. Hence, they had the chance to decide whether their transfer would reach the trustor with certainty or not. Finally, we asked subjects a set of additional questions on general dispositions and socio-demographics in a post-experimental questionnaire.

### 2.2. Behavioral Predictions

Assuming pure self-interest the unique subgame perfect Nash equilibrium of our game predicts that the trustee never returns any positive amount. Therefore, the trustor, anticipating this, does not transfer anything. Subjects with reciprocal concerns (Dufwenberg and Kirchsteiger, 2004; Falk and Fischbacher, 2006) may choose to send/return positive amounts. Based on existing evidence from trust games we expect that a substantial amount of subjects decides to reciprocate. More specifically, we expect that some trustees return positive amounts when the back transfer is certain, and weakly increase their back transfer with the amount received.

Given subjects reciprocate, we are interested in the way they behave when self-image relevant factors are varied. We use the model of Bénabou and Tirole (2011) to guide our analysis^3^. A key component of it is that beliefs about one's pro-sociality type are malleable through actions as imperfect recall is assumed. Thus, identity management becomes possible: if the cost of sending a pro-social signal (via performing a pro-social action) is small enough, pro-self types decide to invest in their identity by imitating a pro-social type^4^. Bénabou and Tirole (2011) also allow for the possibility that inferences from actions about one's type are malleable. Such inferential wiggle room exists, if the informativeness of an action about one's self-image is imperfect^5^. Inferential wiggle room allows self-deception: if the salience of a situation is low enough (reducing the signal strength of an action), pro-social types tend to make a selfish choice as the monetary gain outweighs the negative effect on the self-image.

Our experimental design manipulates self-image related aspects at two stages. First, trustees could exploit the fact that the back transfer is not executed for sure in scenarios 2–4^6^. Second, trustees could succumb to the temptation of choosing a scenario that benefits them.

Trustees may use the possible failure of the back transfer as an excuse to return less in comparison to their scenario 1 back transfer. They may tell themselves that their transfer may fail anyways and their choice will not matter for the trustor. Hence, the situations in scenarios 2 to 4 allow trustees with a desire not to appear selfish toward themselves to engage in self-deception. Essentially, their self-serving interpretation of the scenario's risk allows them to be more selfish. Exley (2015) studies choices between a certain amount and risky lotteries. She varies the recipient of both (self vs. a charity) and finds evidence of the use of risk as an excuse to give less. Haisley and Weber (2010) find such a self-serving bias caused by uncertainty in a related study involving dictator game choices under ambiguity, and Garcia et al. (2020) in the context of charitable giving. Also Di Tella et al. (2015) provide evidence for self-serving interpretations and increased selfishness. See Shalvi et al. (2015) and Gino et al. (2016) for overviews of self-serving justifications, respectively motivated reasoning, used in the domain of ethical/moral behavior^7^. Thus, we expect that some trustees engage in self-deception when they make the back transfer choice. Moreover, the effect of uncertainty should be more pronounced the lower the implementation probability of the back transfer is. Thus, we expect a positive relationship between the probability and their back transfer choice.

**Hypothesis 1**. *In comparison to scenario 1 a non-trivial fraction of reciprocating trustees transfer back less when the transfer could fail (scenarios 2 to 4).*

Bayesian self-signaling would predict the opposite effect on trustees' back transfer choices. A decrease of the transfer's implementation probability cheapens the pro-social signal to the self. Investing in identity becomes more affordable and, in turn, more pro-social choices should result. If trustees engage in identity management, we expect a positive relationship between the probability and their back transfer choice^8^.

**Hypothesis 2**. *In comparison to scenario 1 a non-trivial fraction of reciprocating trustees transfer back more when the transfer could fail (scenarios 2 to 4).*

A second situational excuse arises when subjects are informed that they can choose a scenario themselves. Given equal positive back transfers across scenarios, this choice implies a trade-off between the original scenario 1 that implements the back transfer for sure and a scenario that is favorable to the trustee since the transfer may fail. If this moral wiggle room affects the decision of reciprocators, a substantial amount of reciprocating trustees chooses a scenario that involves uncertainty with respect to the implementation of the back transfer.

**Hypothesis 3**. *When the choice of a scenario that involves uncertainty results in an expected monetary gain, a substantial amount of reciprocating trustees does not choose scenario 1.*

Finally, trustees may also have a desire not to appear selfish to others and, hence, may care about the effect of their choice on the trustor. The positive chance of a transfer failure in scenarios 2–4 allows them to return nothing as the trustor could not distinguish whether getting zero is the consequence of the trustee's choice or due to the failure of the transfer. Thus, returning nothing in scenarios 2–4 is compatible with an image of not appearing selfish to others. The reasoning follows Andreoni and Bernheim (2009) and is also in line with the prediction for the “Probability and Outcome” treatment in Grossman (2015)^9^. The choice of a scenario that involves uncertainty may be due to the manipulation's effect on the desire not to appear selfish toward oneself or toward others. Thus, our design cannot distinguish between the two at this stage. As the instructions do not explicitly mention that the chosen scenario is not communicated to the trustor, we cannot rule out that some trustees falsely believed trustors will be informed about the scenario they chose. This would eliminate the situational excuse (with respect to the scenario choice) for trustees motivated by a desire not to appear selfish to others. Only the situational excuse that affects the desire not to appear selfish toward oneself would remain.

### 2.3. Participants and Procedures

Using the ORSEE software (Greiner, 2004) 128 subjects were recruited among students from various disciplines at the local university^10^. In each session gender composition was approximately balanced and subjects took part only in one session. The experiment was programmed and conducted with the software z-Tree (Fischbacher, 2007) and took, on average, 60 min. The average earnings in the experiment have been €14.17 (including a €2.50 show-up fee).

Upon arrival at the laboratory subjects were randomly assigned to one of the computer terminals. Each computer terminal is in a cubicle that does not allow communication or visual interaction among the participants. Subjects were given time to privately read the instructions and were allowed to ask for clarifications. In order to check the understanding of the instructions subjects were asked to answer a set of control questions. After all subjects had answered the questions correctly the experiment started. At the end of the experiment subjects were paid in cash according to their performance. Privacy was guaranteed during the payment phase.

## 3. Results

Our analysis starts with a big picture look at the effect of transfer level and scenario on trustees' back transfer decisions. We proceed by identifying the subjects who elicit reciprocal concerns. Then, we analyze reciprocators' back transfers across scenarios as well as their scenario choices in order to test how they behave when self-image relevant factors are varied.

### 3.1. Analysis

We first perform random-effects panel regressions with the back transfer as the dependent variable. The panel includes all choices of a trustee (five transfer levels in three/four different scenarios). Standard errors are robust and clustered at the individual level. See Table 1 for results. The specification in column I includes the transfer received and the implementation probability as explanatory variables. Coefficients for both are positive and highly significant. A control dummy for the treatment with four scenarios is not significant.

**Table 1:** Determinants of amount returned.

	**I**		**II**		**III**	
Transfer	1.22^***^	(0.063)	1.22^***^	(0.063)	1.25^***^	(0.062)
Four scenarios	0.76	(0.65)	0.74	(0.65)	0.74	(0.65)
Implementation probability	1.38^***^	(0.49)				
Scenario 2 (90)			−0.25^***^	(0.093)	−0.21^***^	(0.080)
Scenario 3 (80)			−0.40^***^	(0.13)	−0.32^***^	(0.11)
Scenario 4 (50)			−0.69^***^	(0.24)	−0.64^***^	(0.23)
Transfer of 10					−0.099	(0.29)
Transfer of 10 × Scenario 2 (90)					−0.19	(0.16)
Transfer of 10 × Scenario 3 (80)					−0.40^**^	(0.19)
Transfer of 10 × Scenario 4 (50)					−0.22	(0.49)
Constant	−3.56	(2.46)	−2.04	(2.30)	−2.14	(2.29)
Adjusted R^2^	0.46		0.46		0.46	
Observations	2,240		2,240		2,240	

Random-effects panel regression with robust standard errors; in II the scenario with certainty (1) serves as the baseline; significance levels: *** = 1%, ** = 5%, * = 10%.

The specification in column II replaces the implementation probability with dummies for scenarios 2–4. All scenario dummies are negatively correlated with the back transfer. Further tests of the dummy coefficients show that back transfers in scenario 3 and 4 are lower than back transfers in scenario 2 (*p* = 0.055, *p* = 0.058) but not significantly. Also back transfers in scenario 4 are not significantly lower than back transfers in scenario 3 (*p* = 0.129). Overall, there is a positive correlation between the trustor's transfer and the amount trustees chose to return. On average, subjects reciprocate. Moreover, on average, subjects seem to reduce the amount they send back when the scenario implies uncertainty about the implementation of their back transfer.

In a further specification shown in column III we test whether sending the maximum of 10 has an effect on trustees' behavior. For this purpose we add a dummy variable for a transfer of 10 as well as interaction terms with the three scenario dummies. The interaction between the transfer of 10 dummy and the dummy for scenario 3 is negative and significant at the 5%-level, while none of the other additional regressors is significant. Thus, results do not indicate increased selfishness among trustees when less than the full amount is transferred. A Hausman test validates the choice of a random-effects model over a fixed-effects one (*p* = 0.49). If control variables (age, gender) are included in the regression, they are not statistically significant and the reported results are not affected.

We continue the analysis at the individual level. Following Fischbacher et al. (2001) we categorize subjects based on what they return (given a transfer of 0, 2.5, 5, 7.5, or 10) when they make a choice under certainty (scenario 1), see also Table 2. Eleven subjects do not return anything, ever. The back transfers of 109 subjects are increasing weakly monotonically with the amount received and they are classified as conditional cooperators. Eight subjects elicit a humpback-shaped back transfer pattern. They first increase their back transfers with the amount received, but then decrease them. Our analysis considers conditional cooperators (even if they return only very little) as well as only partially reciprocating subjects (humpback-shaped pattern) as reciprocators. At the end of our analysis we will test for the robustness of our results, if humpback-shaped and selfish reciprocators are excluded.

**Table 2:** Categorization of subjects' scenario 1 back transfers.

**Type**	**Number of**	**Mean of returned amount when receiving**				
	**subjects**	**0**	**2.5**	**5**	**7.5**	**10**
Purely selfish	11	0	0	0	0	0
Conditional cooperators	109	0.27	4.02	7.53	10.99	14.8
Humpback-shaped	8	0	4	6.37	7.87	3.87

What is reciprocating trustees' *behavior across scenarios*? More specifically, how did they behave in scenarios 2–4, that is, when there is a positive probability that their back transfer could fail? Table 3 reports the percentage of reciprocating subjects who returned less/same/more in scenarios 2–4 (compared to scenario 1) for each amount received^11^. We perform Wilcoxon signed-rank tests for each transfer level of scenarios 2–4 in order to compare reciprocating subjects' choices under uncertainty to their scenario 1 choices. The majority of subjects does not change the back transfer, yet there is a general tendency to return less under uncertainty. For a relatively high chance of transfer success (90%, scenario 2) the tendency to decrease the back transfer is only significant (at the 5%-level) for amounts received of 2.5 or 5. For an 80% chance of transfer success (scenario 3) the tendency to decrease the back transfer is significant (at least at the 5%-level) for all amounts received except 0. In scenario 4 (implementation probability 50%) the proportion of subjects who decrease is significant (at least at the 5%-level) for all amounts received.

**Table 3:** Pairwise comparison of reciprocating subjects' back transfers.

**Amount received**	**0**	**2.5**	**5**	**7.5**	**10**
Scenario 2 (90%)	6/90.6/3.4	16.2/77.8/6**	16.2/76.9/6.9**	17.1/71.8/11.1	13.7/78.6/7.7
Scenario 3 (80%)	7.7/89.7/2.6*	24.8/67.5/7.7***	27.4/60.7/11.9***	27.4/60/13.6**	23.1/65.8/11.1**
Scenario 4 (50%)	7/93/0**	29.8/57.9/12.3**	33.3/56.2/10.5***	31.6/54.4/14**	31.6/57.9/10.5***

In each cell x/y/z indicates the percentage of reciprocating subjects who returned less/same/more in the respective scenario in comparison to scenario 1. There are 117 reciprocators in scenarios 2 and 3, 57 in scenario 4. Significance of Wilcoxon signed-rank tests of choices under uncertainty compared to scenario 1 choices: *** = 1%, ** = 5%, * = 10%.

We proceed to categorize subjects based on their choices across scenarios. For this purpose we compute, for every transfer level, the difference between back transfers in scenario 1 and 2, 2 and 3, and, if applicable, 3 and 4. The sum of these partial differences expresses how a subject reacted to the variation of the transfer implementation probability. We distinguish between three different behavioral patterns. Some trustees decreased their back transfers with the likelihood that the transfers fails. For each transfer level some trustees returned the same amount independently of the scenario. Finally, some increased their back transfers the more probable it gets that their transfer does not get implemented. Table 4 provides frequencies of these behavioral patterns. The categories appear to be similarly represented in sessions with three and four scenarios. A χ^2^ test (*p* = 0.63) does not reject that the distribution of types is the same. Out of 128 subjects (all sessions pooled), 11 never return anything, 41 decreased, 55 did not change and 21 increased the back transfer across scenarios^12^. Table 4 also reports the mean aggregate back transfers in scenario 1 of each category, that is, the sum of the five back transfer choices. Aggregate back transfers under certainty are not significantly different across categories. Moreover, reciprocators' transfer choices as trustor do not differ across categories (4.88, 4.73, and 4.76), while the ones of purely selfish trustees are significantly lower (2.5).

**Table 4:** Categorization based on the back transfer schedules across scenarios.

	**Total**	**Back transfers**	**Decreased amount**	**Returned same amount**	**Increased amount**
	**subjects**	**always 0**	**across scenarios**	**in all scenarios**	**across scenarios**
3 scenarios	64	4	21	30	9
4 scenarios	64	7	20	25	12
All	128	11	41	55	21
Aggregate back transfers	128	0	36.29 (2.61)	36.18 (2.08)	38.05 (3.71)
in scenario 1,					
mean (st. error)					
Transfer choice	128	2.5 (1.21)	4.88 (0.33)	4.73 (0.41)	4.76 (0.29)
as trustor,					
mean (st. error)					

**Figure 1:**
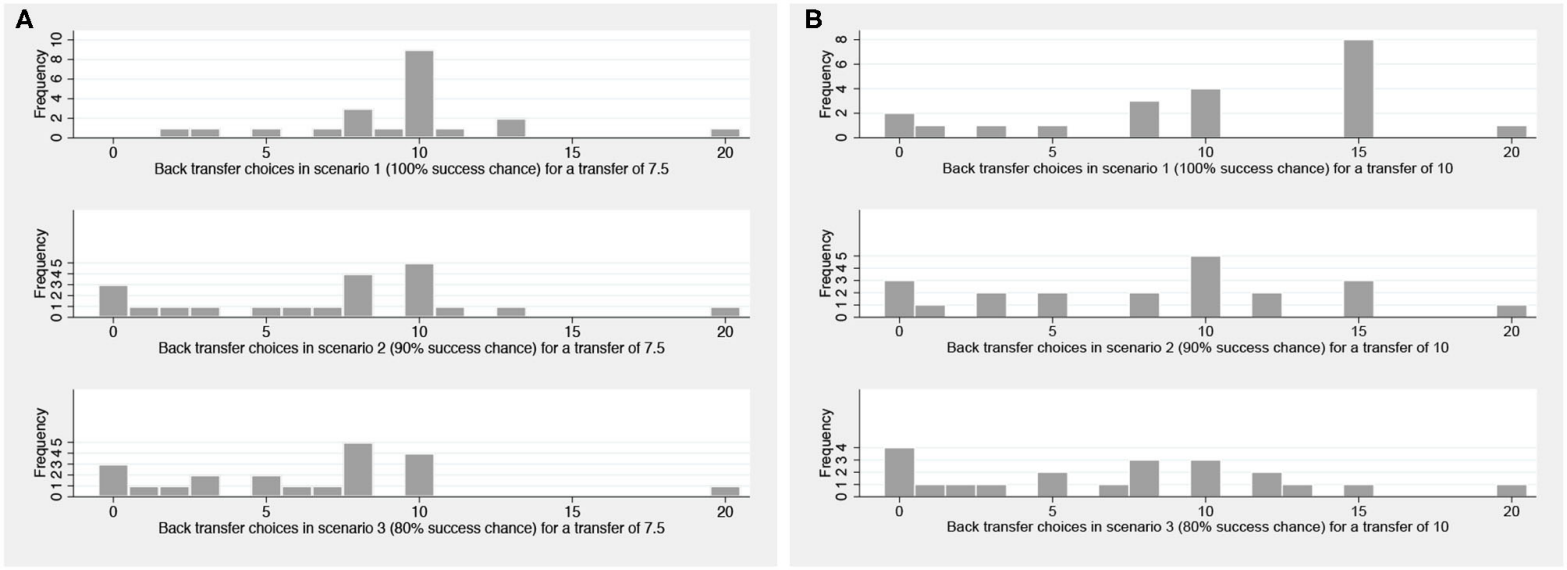
Histograms of back transfers for scenarios 1–3 (for subjects who decreased the amount). **(A)** for a transfer of 7.5 and **(B)** for a transfer of 10.

Reciprocating trustees' behavior across scenarios indicates that 41 subjects reduced their back transfers with the likelihood that the transfers fail. Did these subjects tend to return zero with a positive failure probability or did they make use of the excuse in a more subtle way? Overall, the majority seems to return only slightly less, although few subjects drop their back transfer to zero in uncertain scenarios. Figure 1 shows histograms of back transfers for scenarios 1 to 3 for a transfer of 7.5 (Figure 1A) and 10 (Figure 1B). It serves to illustrate the behavioral pattern among subjects who decreased their back transfers. Under certainty, given a transfer of 10 returning 15 corresponds to sending back half of what has been received and this is the most popular choice of trustees. In scenarios 2 and 3, the number of trustees returning 15 drops sharply and smaller back transfers become more common. The number of subjects returning zero increases when the failure chance of the back transfer is positive, but also those of subjects who decide to return less. We observe a similar pattern for a transfer of 7.5.

The way reciprocators handle the variation of the back transfer success rate across scenarios has implications for our analysis of the *scenario choice*. For subjects who returned the very same positive amount independently of the scenario, being able to pick a scenario unambiguously creates moral wiggle room. A subject who increased transfers across scenarios may have done so in order to invest in identity, thus conveying a signal of being pro-social. In such a case, being in a position to select a scenario with an implementation probability <1 may not be advantageous for the subject's expected utility. Finally, subjects who decreased amounts may have already exploited moral wiggle room when they made their back transfer choices in scenarios with uncertainty. They would only benefit from these choices by actually picking a scenario with an implementation probability <1. It is not clear how they would react to a “second serving” of moral wiggle room, though^13^. Hence, our analysis of the scenario choice focuses on the 55 subjects who did not vary the back transfers across scenarios.

**Figure 2:**
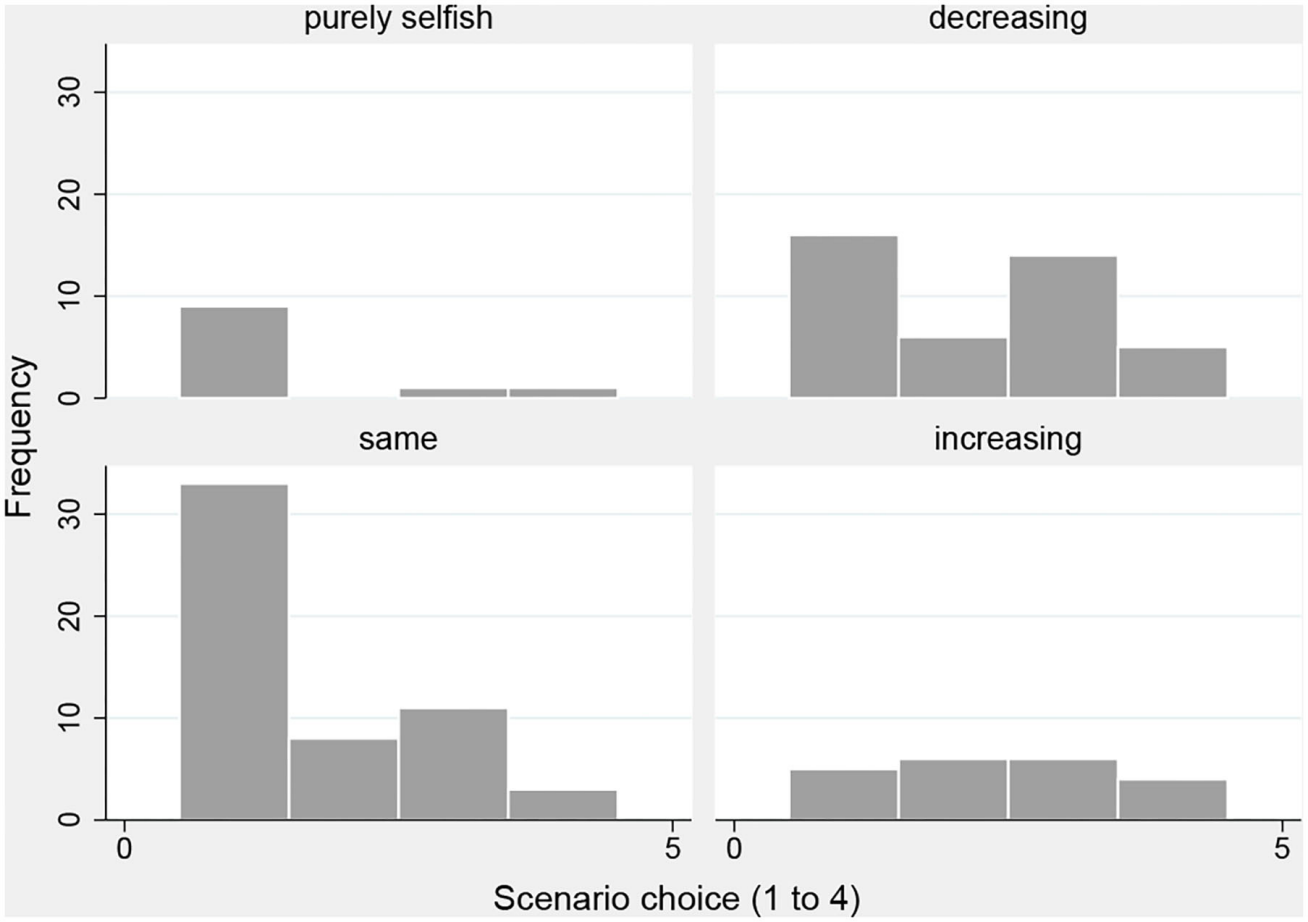
Scenario choices by categories.

Figure 2 shows histograms of the scenario choice for the four categories: purely selfish, decreasing, same, and increasing back transfers across scenarios. For trustees who returned the same amounts across scenarios (Figure 2, bottom left) the scenario choice involved the unambiguous opportunity to reap a monetary gain (in expectations). In this category, 33 of 55 subjects selected scenario 1. In contrast, 22 of them made use of the moral wiggle room and picked a scenario that did not guarantee the back transfer. Allowing for some noise in the decision making (i.e. some trustees, say 25%, select a scenario other than 1 by chance), a one-sided binomial test confirms that this fraction is significantly greater than the noise level (*p* = 0.01) and supports hypothesis 2. When subjects increased back transfers across scenarios, their choice of the scenario should not matter to them and we may expect a uniform distribution. This seems to be the case (Figure 2, bottom right), χ^2^ tests for three scenarios (*p* = 0.67) and four scenarios (*p* = 0.57). Subjects who decreased amounts (Figure 2, top right) appear to have already exploited moral wiggle room when they made their back transfer choices in scenarios 2 to 4. No clear pattern with respect to their scenario choice seems evident. Finally, the scenario choice of purely selfish subjects (Figure 2, top left) has no consequence for their payoffs.

### 3.2. Discussion

In our experiment, 41 reciprocating subjects decreased their back transfer when the failure chance of the transfer was positive, an indication that they made use of this situational excuse. However, 16 of them eventually made a scenario choice that is clearly disadvantageous to them (in expected payoffs terms), while 25 selected a scenario that favors their expected payoffs. Out of 55 reciprocators who returned the same positive amount independently of the situation 22 selected a scenario that implied a positive chance that the back transfer fails to reach the trustor. The remaining 33 selected scenario 1 and made sure the back transfer reaches the trustor. They made no use of moral wiggle room in the scenario 2–4 back transfer choices and resisted the moral wiggle room provided by the scenario choice. Finally, 21 reciprocators increased the back transfer across scenarios, thus, resisting our first and evading our second manipulation. Summarizing, 47 of 117 (40%) reciprocators exploited moral wiggle room, while 70 (60%) resisted (to some extent)^14^.

The back transfer choice across scenarios may be affected by two self-signaling channels and in our experiment we find evidence for both behavioral patterns^15^. However, behavior consistent with self-deception is more common as 41 subjects seem to engage in it compared to 21 whose behavior is consistent with identity management.

If the transfer choice was binary, as in the model of Grossman (2015), identity management predicts low pro-sociality types take the pro-social choice given the cost of the signal cheapens sufficiently. High types are not expected to change their behavior. They already take the pro-social choice under certainty and cannot improve on that. Alternatively, uncertainty about implementation of the back transfer would trigger self-deception processes as the self would perceive the situation as an excuse to behave more selfishly. In a binary context, high types would engage in self-deception (if the psychological cost is small enough), while low types already take the selfish choice under certainty. This implies that in a binary setting the direction of the effect of *p* would depend on the prevalence of low/high pro-sociality types. By design, only low types can invest in identity and self-deception is exclusive to high types. Consequently, identifying either of the behavioral pattern requires sufficient low/high types in the role of the decision maker.

In our experiment, subjects have more than two transfer options to choose from leaving both types the theoretical possibility to go either way. Unless subjects choose an extreme in scenario 1, they can adjust their transfer in both directions under uncertainty. Nevertheless, low types presumably have a higher tendency to respond with identity management and, likewise, high types are more prone to engage in self-deception. However, in our data we do not detect significant differences in the average scenario 1 back transfer across subjects who increase/decrease back transfers under uncertainty. It seems that identity management is not limited to low pro-sociality types and low as well as high types engage in self-deception.

Finally, we discuss the robustness of our results in terms of the design choices we made. It is known that the use of the strategy method may encourage reciprocal behavior due to experimenter demand effects (Zizzo, 2010). In fact, we find more conditional cooperation (reciprocators) among our subjects than in Fischbacher et al. (2001), yet still within the range of results in similar studies. However, there is no indication that our within subjects variation of the scenario biases behavior in any particular way^16^. Moreover, it is worth to note that two of our design choices made our experiment a tougher test environment for moral wiggle room to prevail than comparable experiments. First, while the side-by-side interface for entering back transfer schedules makes it easier for subjects who would like to enter the same positive amounts across scenarios to do so, it may become more difficult for subjects who have a tendency to engage in self-deception to actually do so. Since scenario 1 choices are still visible, the context of the choices under uncertainty is more salient than without the reminder. Second, we let our subjects play both roles which means that trustees are familiar with the trustor's perspective of the situation. This potential awareness about the other role may make it harder to exploit moral wiggle room in comparison to a design in which subjects only play one role.

Last but not least, we would like to stress that our implemented design does allow us to compare behavior consistent with self-deception and behavior consistent with identity management. However, it does not contrast a treatment in which self-deception is possible with a treatment that rules out self-deception. Likewise, it does not feature a treatment in which identity management is not possible. Consequently, our design is able to identify whether one effect dominates the other (at the individual level). It does not quantify the net effect of self-deception, respectively, identity management, though. In a similar design as ours, further treatments could serve as benchmarks to test the prevalence of behavior consistent with self-deception (identity management) against. More specifically, in such a self-deception only treatment the trustee would always have to pay the back transfer—independently of *p*—while the trustor may not receive the back transfer. This would take the chances of possibly benefitting from uncertainty off the table. In an identity management only treatment, the trustor would always get the back transfer, while there is a positive probability that the trustee does not have to pay the back transfer. This remains for future research.

## 4. Conclusion

We conducted a modified trust game in order to analyze how reciprocators respond to systematic changes of self-image relevant factors. In our experiment a substantial amount (40%) of reciprocating subjects behaved less pro-social when we introduced moral excuses for selfish behavior. That is, when the context of their choice became less salient, they succumbed to the temptation of keeping more.

This behavioral pattern is particularly interesting for the trustees' back transfer choices. Uncertainty about implementation of the back transfer may not only be perceived as a situational excuse to behave more selfishly (self-deception) but may also be interpreted as an opportunity to invest in a pro-social self-image (identity management). The two predicted effects go in opposing directions. Our results show that twice as many subjects decrease than increase their transfers under uncertainty. It seems that self-deception is prevalent when subjects make the back transfer choice. However, some trustees do increase their back transfers with more uncertainty about the implementation. It appears that self-image concerns have an ambiguous nature, in the sense that self-signaling processes can go either way: via self-deception they can lead to less giving, via identity management they can induce more giving.

Are there characteristics that distinguish individuals who are prone to self-deception from those who may invest in identity? It seems reasonable to assume that individuals who give more (pro-social types) are more likely to engage in self-deception, while those who give less (pro-self types) tend to be generous in order to boost their ego. However, our analysis does not provide support for this. Self-deceiving behavior and identity management are both used across the entire spectrum of scenario 1 back transfers.

Finally, our evidence also suggests that the effect of situational excuses extends beyond the setting of a dictator game where it has been established so far to the one of a trust game. It seems that the preference to reciprocate is also affected by the availability of situational excuses, just as the preference to give. See also Malmendier et al. (2014) and Regner (2018) for similar findings, while van der Weele et al. (2014) find no effect of a moral wiggle room manipulation in the context of reciprocity. Note, however, that our use of the strategy method—a design feature motivated by being able to test self-deception vs. identity management—can be seen as a relatively weak reciprocity environment (Casari and Cason, 2009). Although our analysis considers only subjects who do actually reciprocate, the direct response method would be regarded as a stronger setting to induce reciprocity.

## Data Availability Statement

The raw data supporting the conclusions of this article will be made available by the authors, without undue reservation.

## Ethics Statement

The studies involving human participants were reviewed and approved by Max Planck Institute of Economics, Jena. The participants provided their written informed consent to participate in this study.

## Author Contributions

All authors listed have made a substantial, direct and intellectual contribution to the work, and approved it for publication.

## Conflict of Interest

The authors declare that the research was conducted in the absence of any commercial or financial relationships that could be construed as a potential conflict of interest.

## Publisher's Note

All claims expressed in this article are solely those of the authors and do not necessarily represent those of their affiliated organizations, or those of the publisher, the editors and the reviewers. Any product that may be evaluated in this article, or claim that may be made by its manufacturer, is not guaranteed or endorsed by the publisher.

## Footnotes

^1^Also van der Weele and von Siemens (2020) test Bayesian self-signaling experimentally but find no empirical support. Grossman and van der Weele (2017) develop a Bayesian self-signaling model in the context of information acquisition where individuals can willfully ignore to get informed. Their related experimental test supports the model.

^2^Results of existing studies which analyze reciprocal behavior when there is a situational excuse for not giving point in different directions. van der Weele et al. (2014) apply the ‘plausible deniability' treatment from Dana et al. (2007) to second-mover behavior in a trust/moonlighting game. They find no behavioral differences in comparison to a baseline and conclude that moral wiggle room has no effect on the incidence of reciprocal behavior. Regner (2018) find a significantly higher rate in three treatments that feature moral wiggle room manipulations (between 37.5 and 45%) compared to the baseline rate of selfish choices (6.25%). Malmendier et al. (2014) analyze an “exit option”—that is, to what extent subjects are willing to avoid an allocation choice even if it is costly—in the context of reciprocity. They find that subjects do sort out in the context of positive reciprocity but sorting out is significantly higher without reciprocity. Thus, our study provides an additional data point.

^3^Several approaches exist to model self-image concerns. See also the literature on cognitive dissonance (Festinger, 1962; Aronson, 1992; Beauvois and Joule, 1996; Konow, 2000; Spiekermann and Weiss, 2016), identity (Akerlof and Kranton, 2000), self-impression management (Murnighan et al., 2001), self-concept maintenance (Mazar et al., 2008) and other self-signaling models (Bodner and Prelec, 2003; Bénabou and Tirole, 2006; Tirole et al., 2016).

^4^Grossman (2015) focuses on this aspect of self-signaling. He tests his model experimentally in a binary probabilistic dictator game. The transfer's implementation probability is either certain or 1/3. The model predicts that giving in the low probability treatment is higher as the expected outcome-utility cost of the pro-social choice is cheapened. However, his results do not offer conclusive supporting evidence.

^5^See their parameter ν, the informativeness of an action, and their footnote 10. See also Tirole et al. (2016) who endogenize the signal strength of an action by incorporating narratives in their self-signaling model. Such a narrative, provided by others or “self-authored,” may serve as an excuse for immoral behavior. It allows individuals to rationalize an immoral action even though it only contains a minimal degree of subjective plausibility.

^6^Generally, how much trustees return in scenarios 2–4 is not only affected by their social but also by their risk preferences (with respect to their own payoff and the trustor's). Trautmann and Vieider (2012) review the literature on social influences on risk attitudes and find no consistent patterns when risky decisions are taken on behalf of others. Based on a meta-analysis, Polman and Wu (2020) identify sub-domains in which there is an effect size in favor of a risky shift (e.g., non-financial choices, when emotions are involved) or a cautious shift (e.g., medical choices, decisions involving children) when people choose for others.

^7^Note that uncertainty does not generally lead to more selfishness. Engel and Goerg (2018) find that dictators' transfers increase with the risk their recipient receives their endowment (expected values remaining equal).

^8^Note that an alternative motivation to increase transfers could be the desire to make sure that the same expected amount reaches the trustor, independently of the scenario.

^9^Note, however, that in our design we cannot be completely sure that trustees assume the trustor will be informed about the possibility that the transfer may fail when trustees make their back transfer choice. They may assume that trustors only learn the outcome. In that case, social-signaling does not predict an effect of a variation of the implementation probability on the incidence of zero back transfers. See Grossman (2015) for the formal argument.

^10^Pre-registering was not yet common when our study was conducted. However, we adhere to the principles proposed by Simmons et al. (2011) to tackle the problem of false-positive publications. With respect to the sample size, we decided ex-ante to collect observations from 4 sessions with 32 subjects each.

^11^One subject selected the maximum back transfers in scenario 1, excluding them from returning more in other scenarios.

^12^The categorization aggregates over choices at all five transfer levels. Hence, it could be that a subject's behavior is inconsistent across transfer levels. One out of 55 subjects categorized as returning the same amount did in fact decrease the back transfers by 2.5 at a transfer level of 2.5 and increased them by 2.5 when receiving 5. All others never deviated from their scenario 1 back transfers. Among subjects categorized as increasing the amount two slightly decreased their back transfer at a transfer level of 0. All others never lowered the back transfer. Out of 41 subjects categorized as decreasing the back transfers one subject increased the amount returned at a transfer level of 5 and one subject was inconsistent. All others never increased the back transfer. If selfish and humpback-shaped reciprocators are considered, 21 subjects are categorized as selfish, 33 decrease, 47 return the same and 19 increase the amount.

^13^They may consciously choose the scenario that maximizes their expected payoff. However, having to pick a scenario that clearly favors them may be too much to still appear pro-social. Then, a choice of a less favorable scenario would result. Their choice might also be affected by moral balancing keeping them from engaging in self-deception two times in a row.

^14^When excluding humpback-shaped and selfish reciprocators (mean of aggregate back transfers in scenario 1 <20), 60 out of 99 (61%) resisted.

^15^An anonymous reviewer from a previous journal submission pointed us to another potential situational excuse. Trustees may think their trustor does not deserve to receive a back transfer, if the trustor's transfer is less than the maximum of 10. Our data do not indicate such a bias, though.

^16^We cannot exclude, though, that presenting the scenarios in the same order may have affected subjects' choices.

## References

[B1] Akerlof G. A. Kranton R. E. (2000). Economics and identity. Q. J. Econ. 115, 715–753. 10.1162/003355300554881

[B2] Andreoni J. Bernheim B. D. (2009). Social image and the 50-50 norm: a theoretical and experimental analysis of audience effects. Econometrica 77, 1607–1636. 10.3982/ECTA7384

[B3] Aronson E. (1992). The return of the repressed: dissonance theory makes a comeback. Psychol. Inq. 3, 303–311. 10.1207/s15327965pli0304_1

[B4] Beauvois J. Joule R. (1996). A Radical Theory of Dissonance. London: Taylor & Francis.

[B5] Bénabou R. Tirole J. (2006). Incentives and prosocial behavior. Am. Econ. Rev. 96, 1652–1678. 10.1257/aer.96.5.1652

[B6] Bénabou R. Tirole J. (2011). Identity, morals, and taboos: beliefs as assets. Q. J. Econ. 126, 805–855. 10.1093/qje/qjr002 22073409

[B7] Berg J. Dickhaut J. McCabe K. (1995). Trust, reciprocity, and social history. Games Econ. Behav. 10, 122–142. 10.1006/game.1995.1027

[B8] Bodner R. Prelec D. (2003). Self-signaling and diagnostic utility in everyday decision making. Psychol. Econ. Decis. 1, 105–126.

[B9] Casari M. Cason T. N. (2009). The strategy method lowers measured trustworthy behavior. Econ. Lett. 103, 157–159. 10.1016/j.econlet.2009.03.012

[B10] Dana J. Weber R. A. Kuang J. X. (2007). Exploiting moral wiggle room: experiments demonstrating an illusory preference for fairness. Econ. Theory 33, 67–80. 10.1007/s00199-006-0153-z

[B11] Di Tella R. Perez-Truglia R. Babino A. Sigman M. (2015). Conveniently upset: avoiding altruism by distorting beliefs about others' altruism. Am. Econ. Rev. 105, 3416–3442. 10.1257/aer.20141409

[B12] Dufwenberg M. Kirchsteiger G. (2004). A theory of sequential reciprocity. Games Econ. Behav. 47, 268–298. 10.1016/j.geb.2003.06.003

[B13] Engel C. Goerg S. J. (2018). If the worst comes to the worst: Dictator giving when recipient's endowments are risky. Eur. Econ. Rev. 105, 51–70. 10.1016/j.euroecorev.2018.03.011

[B14] Exley C. L. (2015). Excusing selfishness in charitable giving: The role of risk. Rev. Econ. Stud. 82, 587–628. 10.1093/restud/rdv051

[B15] Falk A. Fischbacher U. (2006). A theory of reciprocity. Games Econ. Behav. 54, 293–315. 10.1016/j.geb.2005.03.001

[B16] Feiler L. (2014). Testing models of information avoidance with binary choice dictator games. J. Econ. Psychol. 45, 253–267. 10.1016/j.joep.2014.10.003

[B17] Festinger L. (1962). A Theory of Cognitive Dissonance, Vol. 2. Stanford, CA: Stanford University Press.

[B18] Fischbacher U. (2007). z-Tree: Zurich toolbox for ready-made economic experiments. Exp. Econ. 10, 171–178. 10.1007/s10683-006-9159-4

[B19] Fischbacher U. Gächter S. Fehr E. (2001). Are people conditionally cooperative? evidence from a public goods experiment. Econ. Lett. 71, 397–404. 10.1016/S0165-1765(01)00394-9

[B20] Garcia T. Massoni S. Villeval M. C. (2020). Ambiguity and excuse-driven behavior in charitable giving. Eur. Econ. Rev. 124:103412. 10.1016/j.euroecorev.2020.103412

[B21] Gino F. Norton M. I. Weber R. A. (2016). Motivated bayesians: Feeling moral while acting egoistically. J. Econ. Perspect. 30, 189–212. 10.1257/jep.30.3.189

[B22] Greiner B. (2004). The Online Recruitment System Orsee 2.0 - *a guide for the Organization of Experiments in Economics*. Mimeo, Department of Economics, University of Cologne.

[B23] Grossman Z. (2014). Strategic ignorance and the robustness of social preferences. Manag. Sci. 60, 2659–2665. 10.1287/mnsc.2014.1989

[B24] Grossman Z. (2015). Self-signaling and social-signaling in giving. J. Econ. Behav. and Organ. 117, 26–39. 10.1016/j.jebo.2015.05.008

[B25] Grossman Z. van der Weele J. J. (2017). Self-image and willful ignorance in social decisions. J. Eur. Econ. Assoc. 15, 173–217. 10.1093/jeea/jvw001

[B26] Haisley E. C. Weber R. A. (2010). Self-serving interpretations of ambiguity in other-regarding behavior. Games Econ. Behav. 68, 614–625. 10.1016/j.geb.2009.08.002

[B27] Hamman J. R. Loewenstein G. Weber R. A. (2010). Self-interest through delegation: an additional rationale for the principal-agent relationship. Am. Econ. Rev. 100, 1826–1846. 10.1257/aer.100.4.1826

[B28] Konow J. (2000). Fair shares: accountability and cognitive dissonance in allocation decisions. Am. Econ. Rev. 90, 1072–1091. 10.1257/aer.90.4.1072

[B29] Larson T. Capra C. M. (2009). Exploiting moral wiggle room: Illusory preference for fairness? a comment. Judgm. Decis. Mak. 4, 467–474.

[B30] Malmendier U. te Velde V. L. Weber R. A. (2014). Rethinking reciprocity. Annu. Rev. Econ. 6, 849–874. 10.1146/annurev-economics-080213-041312

[B31] Matthey A. Regner T. (2011). Do i really want to know? a cognitive dissonance-based explanation of other-regarding behavior. Games 2, 114–135. 10.3390/g2010114

[B32] Mazar N. Amir O. Ariely D. (2008). The dishonesty of honest people: a theory of self-concept maintenance. J. Mark. Res. 45, 633–644. 10.1509/jmkr.45.6.633

[B33] Murnighan J. K. Oesch J. M. Pillutla M. (2001). Player types and self-impression management in dictatorship games: two experiments. Games Econ. Behav. 37, 388–414. 10.1006/game.2001.0847

[B34] Polman E. Wu K. (2020). Decision making for others involving risk: a review and meta-analysis. J. Econ. Psychol. 77:102184. 10.1016/j.joep.2019.06.007

[B35] Regner T. (2018). Reciprocity under moral wiggle room: Is it a preference or a constraint? Exp. Econ. 21, 779–792. 10.1007/s10683-017-9551-2

[B36] Shalvi S. Gino F. Barkan R. Ayal S. (2015). Self-serving justifications doing wrong and feeling moral. Curr. Dir. Psychol. Sci. 24, 125–130. 10.1177/0963721414553264

[B37] Simmons J. P. Nelson L. D. Simonsohn U. (2011). False-positive psychology: undisclosed flexibility in data collection and analysis allows presenting anything as significant. Psychol. Sci. 22, 1359–1366. 10.1177/0956797611417632 22006061

[B38] Spiekermann K. Weiss A. (2016). Objective and subjective compliance: a norm-based explanation of ‘moral wiggle room'. Games Econ. Behav. 96, 170–183. 10.1016/j.geb.2015.11.007

[B39] Tirole J. Falk A. Bénabou R. (2016). Narratives, Imperatives and Moral Reasoning. Technical report, Mimeo.

[B40] Trautmann S. T. Vieider F. M. (2012). Social influences on risk attitudes: applications in economics, in Handbook of Risk Theory (Dordrecht: Springer), 575–600.

[B41] van der Weele J. J. (2014). Inconvenient Truths: Determinants of Strategic Ignorance in Moral Dilemmas. Available at SSRN 2247288.

[B42] van der Weele J. J. Kulisa J. Kosfeld M. Friebel G. (2014). Resisting moral wiggle room: how robust is reciprocal behavior? Am. Econ. J. 6, 256–264. 10.1257/mic.6.3.256

[B43] van der Weele J. J. von Siemens F. A. (2020). Bracelets of pride and guilt? an experimental test of self-signaling. J. Econ. Behav. Organ. 172, 280–291. 10.1016/j.jebo.2020.02.001

[B44] Zizzo D. J. (2010). Experimenter demand effects in economic experiments. Exp. Econ.13, 75–98. 10.1007/s10683-009-9230-z

