# Supplementary figures and images for: Actions and the Self: I Give, Therefore I am?
Source: Front Psychol. 2021 Aug 10;12:684078. doi: 10.3389/fpsyg.2021.684078 (PMC8382956; doi:10.3389/fpsyg.2021.684078)

## APPENDIX

### A. Screenshots of the Decision Interface

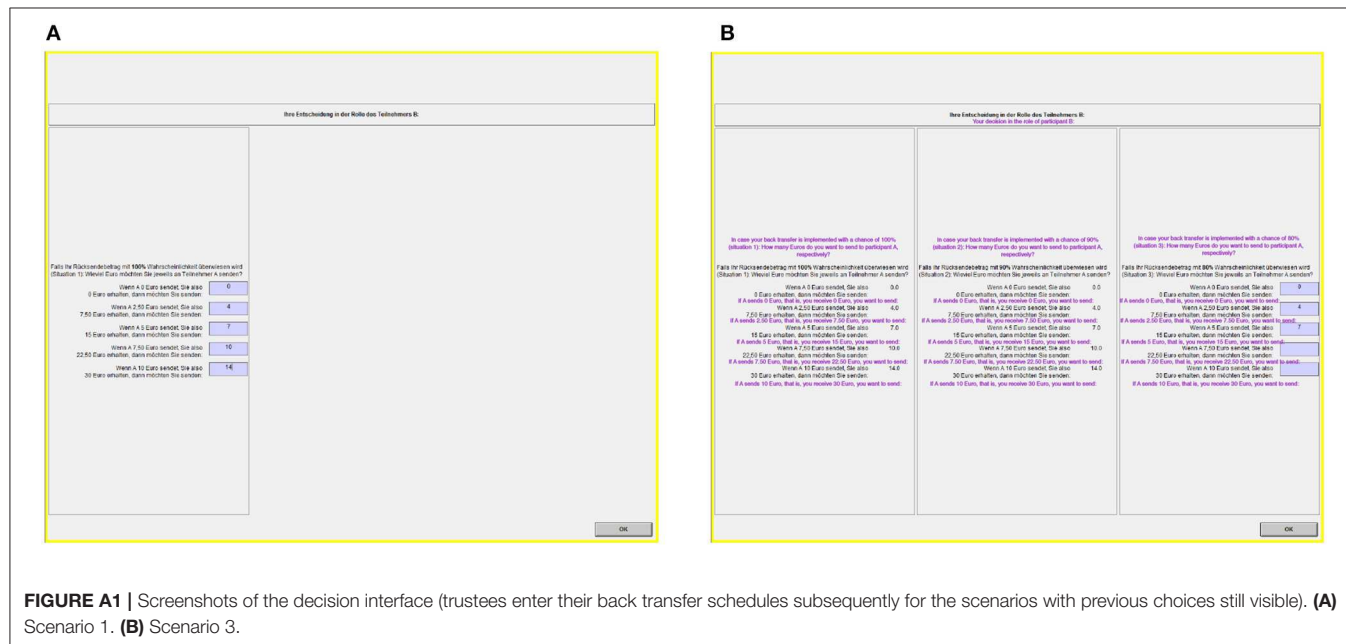

Supplement: Supplementary file 1 [file Data_Sheet_1.pdf]
