# Supplementary material for: Actions and the Self: I Give, Therefore I am?
Source: Front Psychol. 2021 Aug 10;12:684078. doi: 10.3389/fpsyg.2021.684078 (PMC8382956; doi:10.3389/fpsyg.2021.684078)
Supplement: Supplementary file 2 [file Data_Sheet_2.pdf]

Instructions

Welcome and thank you for participating! In this experiment you can earn a monetary amount depending on your decisions and the decisions of the other participants. **Therefore, it is very important that you read the instructions carefully.**

Please note that these instructions are directed to you only and you are not allowed to exchange any information with the other participants.
Also, it is not allowed to talk to other participants during the whole experiment. Whenever you have a question please raise your hand. We will come to your place and answer your question. Please never ask your question(s) aloud. In case you break these rules we will have to end the experiment. Please switch off your mobile phones now.

General procedure

The experiment will take around 60 minutes. Your earnings from this experiment depend on your decisions and possibly on the other participants' decisions. All amounts in the decision situations are stated **in Euro**. The exact amount will be paid to you at the end of the experiment. Additionally, you will receive 2.50 Euro for your participation in the experiment.

After filling out a questionnaire the experiment will be finished and you will receive your payment.

Overview of the procedure:

- reading the instructions, answering the control questions
- decision situations
- questionnaire
- payment and end of the experiment

Details

In the experiment you will be randomly paired with another participant. This means two participants (referred to as A and B) will interact. In the course of the experiment you will take decisions in the role A as well as in the role B. Which role (A or B) will be relevant for your payout will be decided randomly after the experiment. Therefore, it is very important that you **familiarize with both roles**.

Both participants receive an *endowment* of 10 Euro.

First, A will make a decision. He/she can send an amount between 0 and the *endowment* of 10 Euro to participant B. This *transfer* will be **tripled** and given to B.

After that, participant B can decide how much he/she wants to send back to participant A. This *back transfer* can vary between 0 and 10 Euro + 3 * *transfer*.

The following payoffs will result accordingly:

- participant A: *endowment* – *transfer* + *back transfer*
- participant B: *endowment* + 3 * *transfer* – *back transfer*

The following diagram illustrates the game and the resulting payoffs:

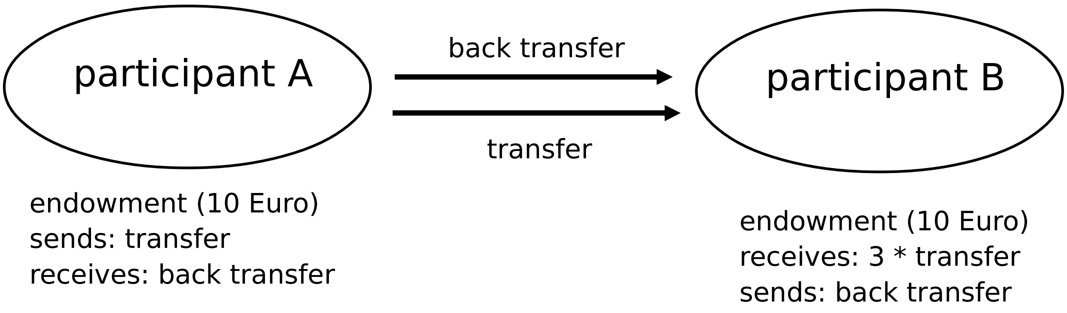

<u>There are 4 different situations for the implementation of the game:</u>

Situation 1: Here, the *back transfer* will be transferred with certainty; this means the *back transfer* will definitely be subtracted from B's account and then added to A's account.

Situation 2: Here, the *back transfer* will be transferred with a 90% chance; thus, the chance of it not being transferred is 10%.

Situation 3: Here, the *back transfer* will be transferred with an 80% chance; thus, the chance of it not being transferred is 20%.

Situation 4: Here, the *back transfer* will be transferred with a 50% chance; thus, the chance of it not being transferred is 50%.

In situations 2 to 4 the following applies:

In case the *back transfer* is transferred, the amount will be subtracted from B's account and then added to A's account. The resulting payoffs are as shown above:

- participant A: *endowment – transfer* + *back transfer*
- participant B: *endowment* + 3 * *transfer – back transfer*

In case the *back transfer* is not transferred, the accounts of A and B will remain unchanged; this means the *back transfer* won't have any impact on the payoffs:

- participant A: *endowment – transfer*
- participant B: *endowment* + 3 * *transfer*

<u>Procedure:</u>

1. Entry of the *transfer* which as participant A you want to send to B. The *transfer* can be chosen from the following values: 0 Euro, 2,5 Euro, 5 Euro, 7,5 Euro, 10 Euro.

2. Entry of the *back transfer*, which as participant B you want to send back to A. You will enter the *back transfer* (in multiples of 10 Cents) for each possible *transfer* (0; 2,5; 5; 7,5; 10). Additionally, you will enter *back transfers* for each of the 4 situations described above.

3. Then, it will be decided which situation occurs. In case situation 2, 3 or 4 is chosen the computer will calculate according to the respective probabilities whether the *back transfers* will be transferred. Afterwards, if applicable the *back transfers* will be transferred.

Payoff

Which role (A or B) will be the relevant role for your final payoff will be decided by chance (50% / 50%). Accordingly, there are two possibilities:

1. You receive your payoff in the role of participant A. In case situation 1 has been chosen your payoff equals *endowment – transfer* + *back transfer*. In case situation 2, 3 or 4 has been chosen your payoff equals *endowment – transfer* + *back transfer* if the *back transfer* is indeed transferred. If it is not transferred your payoff equals *endowment – transfer*.

2. You receive your payoff in the role of participant B. In case situation 1 has been chosen your payoff equals *endowment* + 3 * *transfer – back transfer*. In case situation 2, 3 or 4 has been chosen your payoff equals *endowment* + 3 * *transfer – back transfer* if the *back transfer* is indeed transferred. If it is not transferred your payoff equals *endowment* + 3 * *transfer*.
