# Supplementary material for: Actions and the Self: I Give, Therefore I am?
Source: Front Psychol. 2021 Aug 10;12:684078. doi: 10.3389/fpsyg.2021.684078 (PMC8382956; doi:10.3389/fpsyg.2021.684078)
Supplement: Supplementary file 3 [file Data_Sheet_3.pdf]

Control questions:

The **following example values are not be understood as hints! Please tick the right response (one or more responses can be correct) and fill out all blanks.**

1. There are participants A and B.

A. You only take decisions for the role you randomly have been assigned to (A or B).

B. You take decisions for participant A and for participant B, before you know which role is relevant for your payoff.

C. You take decisions for participant A and for participant B. You already know which role is relevant for your payoff.

D. You take decisions for participant A and for participant B. Both are relevant for your payoff.

2. As participant A you send, for instance, a) 2,50 Euro, b) 5 Euro and c) 10 Euro to participant B.
Participant B thus receives a) _____ Euro, b) _____ Euro and c) _____ Euro.

3. As participant B you receive a) 7,50 Euro, b) 22,50 Euro and c) 30 Euro from participant A. You send back 7 Euro to participant A. Consequently, participant A has a) _____ Euro, b) _____ Euro and c) _____ Euro. You have a) _____ Euro, b) _____ Euro and c) _____ Euro.

4. As participant B you receive a) 15 Euro and b) 22,50 Euro from participant A. You send back 12 Euro to participant A. In situation 1 participant A has therefore a) _____ Euro, b) _____ Euro and you have a) _____ Euro and b) _____ Euro. In situation 2 participant A has with a chance of ___% a) _____ Euro and b) _____ Euro and you have a) _____ Euro and b) _____ Euro. With a chance of ___% participant A has a) _____ Euro and b) _____ Euro and you have a) _____ Euro and b) _____ Euro.

Please raise your hand, if you are done or in case of questions. We will come to you and answer your question(s).
